# Pharmacological Augmentation of Psychosocial and Remediation Training Efforts in Schizophrenia

**DOI:** 10.3389/fpsyt.2017.00177

**Published:** 2017-09-25

**Authors:** Philip D. Harvey, Michael Sand

**Affiliations:** ^1^Department of Psychiatry and Behavioral Sciences, University of Miami Miller School of Medicine, Miami, FL, United States; ^2^Boehringer Ingelheim Pharmaceuticals, Inc., Ridgefield, CT, United States

**Keywords:** schizophrenia, cognition, disability, everyday functioning, pharmacological cognitive enhancement, combination therapy

## Abstract

Pharmacological approaches to cognitive enhancement have received considerable attention but have not had considerable success in improving their cognitive and functional targets. Other intervention strategies, such as cognitive remediation therapy (CRT), have been shown to enhance cognitive performance but have not been found to improve functional outcomes without additional psychosocial interventions. Recently, several studies have attempted to enhance the effects of CRT by adding pharmacological interventions to the CRT treatments. In addition, as CRT has been shown to synergistically improve the effects of psychosocial interventions, the combination of pharmacological therapies aimed at cognition and psychosocial interventions may itself provide a promising strategy for improving functional outcomes. This review and commentary examines the current state of interventions combining CRT and psychosocial treatments with pharmacological augmentation. Our focus is on the specific level of effect of the pharmacological intervention, which could be enhancing motivation, training efficiency, or the consolidation of therapeutic gains. Different pharmacological strategies (e.g., stimulants, plasticity-inducing agents, or attentional or alertness enhancers) may have the potential to lead to different types of gains when combined with CRT or psychosocial interventions. The relative potential of these different mechanisms for immediate and durable effects is considered.

## Introduction

Cognitive impairments are prominent in several neuropsychiatric conditions (1). These impairments are functionally relevant and persistent over time and are minimally related to treatments for the illness (2). These impairments have spurred multiple treatment efforts spanning pharmacological, psychosocial, psychotherapeutic, and rehabilitation-based treatments. The rehabilitation-based treatments have used both in-person and computer-delivered cognitive remediation therapy (CRT) interventions (3). Furthermore, it has been suggested recently that combined pharmacological and cognitive remediation approaches may have the most promise for improving cognitive impairments in severe mental illness generally, and in schizophrenia specifically (4). However, much less attention has been paid to the potential combination of pharmacological treatments with psychosocial interventions. Pharmacological interventions may have the potential to synergistically combine with learning-based psychosocial treatments, much like the combination of these treatments with CRT training.

Previous reviews have analyzed the outcomes of both CRT and pharmacological interventions in schizophrenia and have demonstrated that the former is the more successful of the two. Clearly, cognitive remediation studies have demonstrated greater success, with several different studies finding effect sizes in the moderate to large range (3). In contrast, only a few studies have shown benefit for pharmacological treatments (5) and there is a lack of successful replication of these data and, to date, no positive phase III results have not been confirmed with larger samples in phase III studies (6). Thus, current research with pharmacological interventions have not demonstrated convincing ability to improve cognition as a monotherapy approach.

As the overarching goal of cognition-enhancing treatments is disability reduction, additional treatment approaches are needed to help achieve this. There have been multiple attempts to combine psychosocial interventions with CRT in order to enhance rehabilitation outcomes, but relatively fewer attempts to use the potential parallel strategy of combining targeted cognition-enhancing pharmacological therapies with psychosocial interventions. Most psychopharmacological and disability-targeted treatment data concern the pharmacological factors affecting interfere with the results of psychosocial interventions; however, this review focuses on the possibilities of additional therapeutic intervention.

In this article, we will focus on the combination interventions that could enhance cognitive performance and everyday functioning in people with schizophrenia. Encouraged by several very recent studies, we will examine the characteristics of CRT approaches that are suitable for combination with pharmacological interventions, as well as evaluating pharmacological interventions for their potential for combination with CRT, evaluating a model previously referred to as Pharmacologically Augmented Cognitive Training (PACT) (7). In addition to reviewing the possible benefits of combined CRT and pharmacological interventions, we also consider the possibility that combined pharmacological and psychosocial or psychotherapeutic interventions will have additional benefits when compared to either medication or behavioral intervention alone. The rationale for combining pharmacological cognitive enhancement with a psychosocial intervention is similar to that for adding CRT to a psychosocial intervention: using a synergistic therapeutic approach to enhance cognition through multiple strategies may enhance skill learning.

The evaluation of pharmacological strategies focuses on the mechanism of action, duration of effect, and potential impact on learning-based interventions such as cognitive remediation or behavioral interventions. This evaluation also necessitates a task analysis of CRT interventions, in terms of which cognitive processes are potentially important at different stages of the CRT participation process. Furthermore, available pharmacological interventions may interact with different stages of the CRT engagement process, which will refer to as performance-side variables.

An additional consideration when evaluating pharmacological augmentation of CRT is the fact that some augmentations may also increase the efficacy of CRT. For instance, if the rate of efficiency of training and gains across CRT levels was increased, it might also be the case that near transfer to neuropsychological test performance would be increased as well. There are several different ways that this increase could happen, including increased neuroplasticity (8) which could lead to greater beneficial brain changes with similar levels of effort and achievement.

## Factors Influencing the Efficacy of CRT: Performance-Side Variables

Cognitive remediation therapy has been shown in multiple studies to improve performance on neuropsychological tests, although the efficacy of interventions varies across individuals (9). Several predictive factors for response to CRT have been identified, some of which may also be amenable to benefits from pharmacological interventions, as evaluated below.

There are several factors that can impact on the ability to perform in the training setting, and any of these factors can also influence gains during training. CRT differs from purely pharmacological interventions in that sustained effort-related participation on the part of the trainee is required. This level of effort is sustained for a training session that spans ≥30 min and thus, willingness and ability on the part of the trainee to participate in the training procedure is a prerequisite for training-related gains. While adherence to medication is clearly a participatory activity, the amount of effort expended, particularly in clinical treatment studies where medication is prepacked and delivered to the participant, seems less than that required to train for ≥30 min on a cognitively demanding task, ≥2 times/week.

## Motivation

Motivation has been shown to exert an influence on cognitive changes associated with CRT (10). There are different types of motivation, including motivation induced or maintained by extrinsic factors and motivation arising from intangible and self-generated factors, which is often referred to as “intrinsic” motivation. For instance, several studies by Medalia et al. have documented a positive relationship between intrinsic motivation and CRT outcomes (10–13). Intrinsic motivation refers to the willingness to perform the task because of the perception of benefit, rather than external rewards. The perception of intrinsic benefits is often augmented by certain elements of the CRT training process, such as bridging and discussion groups. It has been hypothesized that provision of external rewards for participating in CRT, such as financial compensation for training sessions, may lead to reduced transfer to other outcomes; however, it has recently been shown in a large-scale study that financial incentives to engage in behavioral rehabilitation programs do not lead to increases in clinician-rated engagement in treatment (14). In this study, individuals who showed high levels of motivation prior to treatment engagement demonstrated the greatest gains, while less motivated individuals did not show increased motivation, even with a potential to obtain considerable financial gain for increased treatment engagement.

### Components of Motivation

#### Global Motivation to Participate in Therapeutic Activities

There are both global and specific components of motivation. Global motivation is the willingness to engage in an activity with the expectation that some benefit will be received. Thus, global motivation may require the ability to understand the means-ends relationships between engagement in remediation interventions and possible real-world gains. Individuals who are unaware that they have either cognitive or everyday functional deficits could therefore be expected to be less likely to be motivated to engage in treatment.

In several previous studies, it has been shown that individuals with schizophrenia who are unaware of their clinical symptoms, or functional or cognitive limitations, are also most likely to report that they have high quality of life and no mood symptoms. For instance, in the large-scale CATIE study, patients who reported that they were “pleased” or “delighted” with their quality of life were rated clinically as having less awareness of their illness (15). Those same patients also reported minimal depressive symptoms, but performed more poorly on tests of executive functioning than patients who had less positive self-assessment of their quality of life.

A recent study has shown the connection between insight and cognitive performance in patients with schizophrenia, as well as improvements in their cognitive performance with treatment. In this study, clinically unstable patients with schizophrenia were recruited as inpatients and treated with lurasidone, quetiapine, or placebo (16, 17). The outcomes measured included ratings of their clinical symptoms as well as performance-based assessments of cognition. At baseline, approximately 33% of patients had performance-based cognition scores that were invalid, presumably because of lack of adequate engagement or effort made in the assessment process. Interestingly, clinical ratings of poor insight into illness were more severe in patients whose test performance was invalid. After antipsychotic treatment, clinical insight improved, and there was a statistically significant improvement in cognitive performance in the trial which was found to be correlated with improvements in insight. Furthermore, patients who provided invalid test performance at baseline were able to generate valid scores after pharmacological treatment.

These data suggest that lack of awareness of impairment may be associated with the inability or unwillingness to engage in cognitive testing. CRT is considerably more demanding than a one-time cognitive assessment, and thus unawareness of impairment clearly has the potential to reduce global motivation to engage in a therapeutic activity. Although there is little research on this topic, these findings also suggest that clinical stability, including the presence of the awareness of clinical, cognitive, and functional status may be a prerequisite for success CRT. As a result, one pharmacological intervention strategy to combine with CRT may be the administration of adequate levels of antipsychotic medications in order to maintain clinical stability and thus potentially augment CRT results.

#### Specific Components of Motivation to Engage in Computerized Training

Cognitive remediation therapy also requires the willingness to interact repeatedly with technology, either in the form of computers or tablet devices. One of the reasons for the success of contemporary CRT interventions is the continuous feedback delivered to the trainee. As most CRT interventions use titrated difficulty, the feedback is positive about 80% of the time on a trial by trial basis. Furthermore, most programs also deliver prizes, messages, and other tokens of achievement, with the goal of feedback being to have the participant find the experience rewarding, hence increasing their level of engagement and motivation to continue to participate. Receipt of tokens and other awards can be considered an extrinsic reward; however, performance feedback also aims to increase the intrinsic motivation to continue to improve in performance.

A further form of encouragement is offered in the form of in-person activities associated with many CRT programs. These activities often take the form of bridging groups, which are meetings designed to make the CRT experience meaningful and useful in real-world cognitively demanding situations. These interventions further aim to offer social support for engagement in a largely technology-oriented intervention. The inclusion of these groups has been argued to be necessary for successful gains in CRT treatment (18). However, it may be the case that, in order to achieve real-world functional gains, the additional intervention needs to be highly relevant to everyday functionality and targeted at those skills. At least two recent studies have shown that CRT combined with bridging groups did not improve performance on either everyday outcomes or on measures of the acquisition of everyday functional skills (19, 20). In one of them (19), CRT plus bridging improved cognitive performance, but not functional capacity or everyday outcomes, whereas CRT plus bridging plus functional skills training improved cognitive performance, functional capacity, and everyday outcomes (20).

There are some illness-specific challenges in schizophrenia, for example, deficits in sensitivity to rewards, both extrinsic and intrinsic, are commonly reported in patients with schizophrenia and may underlie some of the “anhedonic” features of the condition (21). Deficits in social motivation, including active social avoidance, are also common in schizophrenia (22). These motivational deficits have been shown to be more closely tied to social outcomes than neurocognition, social cognition, or even social competence (23–25). Thus, people with schizophrenia may not be sufficiently sensitive to rewards as unaffected individuals, and their social amotivation may also decrease their interest in bridging groups. In terms of extrinsic reward, it has been shown in several recent studies, reviewed by Gold et al. (26), that patients with schizophrenia are less responsive to financial reward for performing tasks than healthy comparison individuals, suggesting that reduced reward sensitivity applies to both tangible and intangible (i.e., social) reward systems.

It has been reported that self-administered CRT training has adherence rates of approximately 70% in both first episode psychosis, and the prodromal phases of the schizophrenia (27, 28). In these two studies, near transfer cognitive gains also appeared to be similar to previous studies that used in-person delivery of CRT, but not formal psychosocial interventions. Assessments to determine whether an individual with schizophrenia has high enough levels of active social avoidance to make coming in for treatment challenging may therefore be appropriate.

## What is the Mechanism of Pharmacological Augmentation of CRT?

Pharmacological interventions have the potential to change motivation through modifying perceived reinforcement. For instance, stimulant medications such as amphetamine and related compounds (e.g., methylphenidate) have the potential to increase sensitivity to reward. As compounds that directly influence the dopamine-mediated reward system, they may increase the reward salience of CRT tokens and awards (29), as well as potentially increasing the sensitivity to treatment gains of an intrinsic nature. They may also increase intrinsic motivation through changing the salience of social interactions, but this is less well-studied than reward augmentation. These compounds also have the potential to improve attentional performance, which has the face-valid implication of increasing the ability to concentrate on training tasks. Further, processing speed may be augmented by these interventions, which can make it easier to make more rapid early gains and solidify motivation to perform.

## PACT Results with Stimulant Medications: is Attention Augmented?

A very recent study has suggested that single-dose amphetamine treatment immediately prior to CRT training leads to increased gains in performance on the training test. Swerdlow et al. treated patients with schizophrenia with 10 mg of amphetamine or placebo in a double blind cross-over design (30). A test for auditory attention processing was administered around before and after 60 min of auditory training. Compared to placebo, these data suggested that amphetamine treatment had a substantial benefit on gains during auditory training, suggesting that session by session administration of cognition-enhancing compounds can lead to greater attentional gains with CRT. Furthermore, the benefit of treatment persisted one week after a single 1-h training session, an effect not seen with placebo.

However, this study does not address the other hypothesis mentioned above; that stimulant treatment may enhance the reward salience of CRT tokens and awards, which could lead to sustained motivation to engage in the task. Also, the motivation to engage in treatment due to perception of long-term gains might also be changed through interventions that increase potential sensitivity to the need to engage in productive activities such as work. This is clearly an important research topic and would require additional research efforts.

Other compounds related to stimulants have also been examined for their augmentation potential. Most commonly, modafinil, an alertness promoting agent, has been studied for both direct cognitive benefits and for augmentation of PACT. The results from modafinil studies have been complex, in that several single-dose studies have reported positive effects of treatment with modafinil as a monotherapy (31), while several multiple dose studies did not find similar efficacy. In a recent 10-session PACT study comparing a standard dose of 200 mg of modafinil to placebo, with the MATRICS consensus cognitive battery (MCCB) as the outcome measure, there was no augmentation effect on CRT (32). Both groups improved in performance, but the performance of the group receiving only CRT was not so substantial that it could have led to a ceiling effect that obscured the effects of modafinil augmentation.

It is of interest, therefore, whether the results of Swerdlow et al. (30), which examined a single dose of amphetamine vs. placebo, would result in the same lack of efficacy if a multiple dose strategy was employed. If the single-dose effect habituates over time, then this would not be an effective intervention. Similarly, no studies have yet examined the consequences of daily dosing with stimulant-like medications on response to CRT interventions. Clearly, this is an area where more research would be important, particularly in terms of the relative benefit of single-dose, multidose day of training, and daily dosing regimens.

## Other Pharmacological Augmentation Strategies

Cognitive remediation therapy has often been described as an intervention which promotes neuroplasticity. Changes in both brain structure and function are commonly noted after CRT interventions, with changes in white matter structure and cognitive activation in response to cognitive stimuli (33). In addition, changes in serum levels of brain-derived neurotrophic factor (BDNF) have been detected following CRT interventions (34). These changes approach full normalization of BDNF in cases treated with active CRT, while cases who participated in video game control treatment did not show these changes, despite equivalent levels of cognitive activity during the treatment period.

Pharmacological compounds too have the potential to lead to changes consistent with neuroplasticity. Compounds affecting the glutamatergic system have been cited as increasing brain plasticity response to various cognitive interventions in animal models (35). These interventions include increases in the rate of learning new information and the extent to which new information is rapidly consolidated in an adaptive manner. Much of this research has focused on facilitation of novel object recognition and maze learning paradigms (36). Both of these processes are also affected by antagonists at glutamatergic sites, including PCP and ketamine (37).

Importantly for this review, glutamatergic agents have been studied extensively in humans in other conditions because of their potential for modifying the learning process. In particular, d-cycloserine (DCS), initially used to consolidate fear extinction gains (38), has been studied for its potential to modify the memory consolidation process (39). This process is hypothesized to occur as a result of partial agonistic effects of DCS on *N*-methyl-d-aspartate glutamatergic receptors. This area of investigation has been challenging because, as DCS causes memory consolidation, it can cause consolidation of both the extinction of the older memories and consolidation of memory recurrence as well (40). Thus, DCS seems at present to have a role in exposure interventions, but the treatment needs to be carefully administered.

There have been multiple studies of the direct effects of various glutamatergic agents on cognition. These results have been consistently negative when using DCS, cycloserine, glycine, and glycine transport inhibitors to improve cognition directly (41, 42). However, this does not mean that these compounds would not have a beneficial effect when combined with CRT interventions.

To date, there has been one study that used DCS in concert with CRT (43), which treated patients with schizophrenia with 50 mg of DCS vs. placebo once weekly, 60 min prior to receiving a CRT session. There was a target of 3 CRT sessions per week over a planned 8-week trial and participants completed an average of 26 training sessions. The results of the trial indicated that participants who were treated with DCS showed improvement on the tasks in the CRT training procedure, but not on external cognitive outcomes, as measured by MCCB, which improved in the placebo group but not the active treatment group. Furthermore, DCS improved negative symptoms in patients who had clinically significant symptoms of this type at baseline, although there was no connection between cognitive gains and negative symptom improvements. Thus, the results of this study are complex; DCS appeared to enhance performance on the training tests, but the fact that patients treated with placebo had greater near transfer of training to cognitive test performance suggests that DCS may interfere with the commonly found transfer of CRT gains to cognitive performance. While improving negative symptoms is an important goal of treatment, this study suggests that improving motivation-related symptoms does not necessarily lead to better ability to engage in cognitive training procedures. The interference with transfer to untrained tests also suggests that DCS may not be the optimal strategy to promote transfer, in terms of both near transfer to cognitive test performance, and far transfer to real world functioning.

An additional study examined the effects of a related pharmacological compound, d-serine, on augmentation of CRT in schizophrenia (44). In this study, it was found that augmentation of CRT treatment did not lead to incremental benefits. However, the authors reported good safety outcomes and suggested that a higher dose may be required.

## Conclusion on PACT

Pharmacological augmentation of CRT has been attempted and some success has been reported for certain study designs, primarily those using stimulant-like drugs. However, daily dosing studies have not yet been published yet, and studies of interventions aimed at increasing the potential neuroplasticity effect of CRT have been less successful to date. Furthermore, all of the possible dosing strategies and augmentation possibilities have not been explored. At this interim stage of the research process, the reasonable conclusion is therefore that this is a developing research area and more studies will most likely be reported in the immediate future which will hopefully enable firmer conclusions to be drawn.

## Psychosocial Rehabilitation and the Interface with Cognitive Enhancement

Psychosocial rehabilitation efforts can be targeted at multiple domains of disability. The two main areas of focus in the past have been social skills (often referred to as social competence) and vocational outcomes, which have both reported some success with psychosocial rehabilitation. For instance, supported employment programs using an individualized placement and support (IPS) model have found that participants who received high quality services had a rate of obtaining competitive employment of approximately 40%, compared to 10–15% for patients who received standard psychiatric rehabilitation services (45). For social skills training, a Cochrane systematic review suggested that average intervention was not more effective than discussion groups for improving social functioning, relapse rates, mental state or quality of life (46). As social cognition is a critical component of social outcomes, there have been multiple attempts to train social skills. These interventions have had several forms, including training focused on social interactions [Social Cognition Intervention Training (SCIT)] and computerized training interventions. Results of studies of SCIT have suggested that there is some moderate benefit on performance-based measures of social cognition, particularly measures of hostile interpersonal interactions, but minimal effects on real-world social outcomes (47–50). Computerized interventions have also shown some promise in terms of improving performance on social cognition measures. For instance, training on the Mind Reading: An Interactive Guide to Emotions (MRIGE) program improved social cognitive performance in individuals with an autism spectrum condition (51).

However, it is clear from the results of psychosocial interventions aimed at social functioning and vocational outcomes that, at most, half of the patients treated show benefit. Interestingly, it has also been shown that when compared to psychosocial interventions alone, patients who receive combined cognitive remediation and psychosocial interventions make more substantial and rapid gains. For instance, McGurk et al. (52) added approximately 20 sessions of CRT to an IPS model vocational intervention and found employment gains that were persistent for 3 years. In a follow-up study, McGurk et al. (53) also found that adding CRT to IPS in IPS non-responders led to a rapid and sustained improvement in employment outcomes, this demonstrating that augmenting cognitive functioning can lead improve the response to ongoing psychosocial intervention.

This CRT enhancement effect appears robust across various psychosocial interventions. Bowie et al. (20) compared monotherapy with skills training using the Functional Adaptation Skills Training (FAST) model developed by Patterson et al. (54) or CRT alone to a combined FAST and CRT intervention, finding that the combined group had significantly greater gains in everyday functioning outcomes than both other treatments, as rated by blinded observers. FAST or CRT as a monotherapy led to domain-specific gains (functional skills and cognition, respectively) but no psychosocial improvements. These results suggest that CRT interventions can successfully combine with skills training interventions that are broadly aimed at functional skills in social, residential, and vocational domains.

Finally, Lindenmayer et al. (55) examined the combination of computerized social cognition training (MRIGE) and CRT compared with CRT alone on changes in performance-based assessments of social cognition and clinician ratings of clinical symptoms and social outcomes in patients with schizophrenia. Their study did not include a monotherapy social cognition arm, as their interest was in whether CRT improved social cognition. Their results indicated that the combined therapy lead to greater gains in performance on social cognitive tests as well as more gains in everyday social functioning without having a symptomatic benefit. Combining MRIGE and CRT did not dilute the effects of CRT on composite cognitive performance, which was significantly improved from baseline in both groups.

## Cognitive Behavior Therapy (CBT) as an Augmentation Target

Another domain of psychosocial interventions aimed at improving symptoms of schizophrenia is that of CBT. CBT interventions are commonly targeted at treatment-refractory delusions or hallucinations. By their definition, CBT interventions require cognitive capacity for efficacy, and as a result, the substantial cognitive impairments seen in schizophrenia, even more salient in patients with evidence of clinical treatment resistance that would lead to CBT intervention, would seem to mitigate against the benefits of a learning-based therapy in patients with major learning problems. In fact, despite evidence of efficacy and considerable enthusiasm for CBT on the part of many proponents, the number needed to treat is higher than that for many pharmacological interventions (56).

Given that CBT interventions are targeted at populations selected for treatment-resistance and increased cognitive impairments, augmentation with CRT aimed at cognition would seem a viable strategy. Interestingly, there seems to be only one published study where CRT was combined with CBT in patients with severe mental illness (57). In that study, patients randomized to CRT prior to treatment with CBT had a more rapid response to CBT than cases randomized to other psychosocial interventions. A similar strategy could also be employed with pharmacological interventions. It would be straightforward to perform a randomized trial combining potential cognition-enhancing medications with CBT in order to see whether there was either faster response or increased benefit.

## Augmentation of Psychosocial Training with Pharmacological Interventions

The substantial successes recorded from the combination of CRT and psychosocial interventions raise the question as to whether pharmacological augmentation strategies could lead to similar gains. While most pharmacological strategies have not had success on their own in terms of enhancement of cognitive performance, the combination of pharmacological interventions with CRT described above has led to incremental gains in some studies. Psychosocial interventions are themselves cognitively active and the possibility exists that synergistic effects could be seen with the combination of pharmacological augmentation of psychosocial interventions.

One interesting study has suggested that pharmacological factors may be critical for skills training interventions (58). It has been known for years that anticholinergic treatment of patients with schizophrenia is correlated with memory impairments. Memory impairments are functionally relevant, being one of the impairments most strongly correlated with everyday functional deficits. Even more important is the finding that anticholinergic medication levels correlate with the efficacy of CRT interventions. Specifically, Vinogradov et al. reported that serum levels of anticholinergic medications shared 20% of the variance with improvements in cognitive performance associated with CRT training. Thus, higher levels of anticholinergic medication can lead to more than just cross-sectional cognitive impairments, they can actually constrain the extent to which CRT provides a beneficial effect.

In another, recent study, similar effects were found for the influence of anticholinergic treatment and psychosocial treatments. Seventy patients with schizophrenia enrolled in psychosocial interventions were followed for 3 years (59). Total anticholinergic burden was assessed and patients were examined for cognitive performance and for progress in their psychosocial intervention programs. Anticholinergic burden predicted cognitive performance on the MCCB, which in turn predicted progress in rehabilitation. Clinical symptoms, antipsychotic treatments, and baseline level of functioning did not add variance to the MCCB scores for prediction of rehabilitation outcome. These data provide convincing evidence for the direct adverse effects on skills training of anticholinergic medications.

## Procholinergic Treatments for Augmentation of Skills Training

Directly in line with the idea that that the cholinergic system may be critical for successful CRT and psychosocial intervention is the idea that procholinergic treatments may be a reasonable pharmacological enhancement strategy. There are two different approaches to procholinergic treatment: treatment with compounds that affect the muscarinic cholinergic system and others that target the nicotinic cholinergic system. Muscarinic targets have included M1 agonists and, much more commonly, acetylcholinesterase inhibitors (AChEIs). Nictonic targets have included a variety of partial agonist strategies aimed at the α-7 receptor with a smaller number of studies targeting a different receptor complex the α4β2.

Results from studies of the direct effect of cognitive enhancement with AChEI have been consistent and disappointing. Three different AChEI (donepezil, galantamine, and rivastigmine) have shown preliminary success in small scale studies, but larger studies have been consistently negative (60–62). In fact, in one study, placebo treatment was superior to active treatment with donepezil (62). Another large-scale study examining galantamine (61) showed some that there were some domains of cognition that were potentially beneficially affected, but the overall effects of treatment on the predetermined cognitive outcomes were negative.

There have been other studies in patient with schizophrenia using the M1 agonist xanomeline (63). Although the compound appears to have efficacy for cognitive performance, its original manufacturer stopped its development because of significant gastrointestinal distress which lead to a substantial rate of discontinuation. At the present time, there are efforts to bypass the toxic effects of M1 agonist compounds by attempting to deactivate the mechanisms responsible for some of the side effects of the treatment.

Results with α4β2 treatments have also been reported. In a substantially powered randomized trial, the α4β2 compound varenicline was found to work as an effective smoking-cessation treatment, but did not have any detectable cognitive benefits, as measured by MCCB (64).

There has been much a more substantial effort in the domain of α-7 receptor agonists; however, results have been inconsistent. Several different treatments have failed to show differences from placebo in studies on patients with schizophrenia (65, 66). A short-term study of the α-7 receptor agonist DMXB-A found separation between active and placebo treatment in a cross-over design (67). However, a longer study with more participants found no beneficial effects of treatment (68). There were reports of substantial successes in a phase II clinical trial, which also had suitable coprimary measures (69). However, this drug also failed to separate from placebo in a much larger-scale phase III study of patients with schizophrenia (Hilts et al., in preparation). Finally, in a much smaller study, the mixed α-7/α4β2 receptor agonist tropisetron was reported to improve cognition in three different samples of 10 patients with schizophrenia compared with placebo (70). However, given the repeated failures of larger studies in this research area to replicate the results of smaller studies and the fact that tropisetron has been in clinical use for two decades for smoking cessation, a much larger confirmatory study will be needed.

The failures of procholinergic agents as monotherapy for cognition in schizophrenia do not necessarily mean that they would not be effective in improving the efficacy of either CRT or psychosocial interventions. Indeed, there is a precedent for a specified combination of a pharmacological agent and a psychosocial intervention: varenicline as an adjunct to clinical interventions for smoking cessation. Although varenicline may lead to reduced smoking in some populations, it has not been approved as a monotherapy. Thus, it is entirely possible that a combination of a pharmacological agent not approved on its own and either a CRT or psychosocial intervention could happen in the future.

Testing such interventions would seem to be a priority but may be challenging while funding agencies insist that all interventions targeted at severe mental illness have an identifiable, separable, and discretely measurable target to engage. In fact, the most recent developments in the “precision medicine” initiative of the National Institute of Mental Health (NIMH) have argued for an identifiable, single target and clear specifications of what constitutes a negative result. While neuropsychological tests can be easily specified as the targets for both pharmacological agents and CRT, the “target” for skills training may be more difficult to define. While Bowie et al. (20) used performance on a measure of functional capacity (the UPSA-B) as the index of treatment gains associated with a highly specific skills training program, vocational interventions are more challenging. While no one would argue that increases in hours worked or money earned is not important, it easy to criticize on the basis that there are multiple potential mechanisms of influence that could move these outcomes in a positive direction. Thus, the combination of pharmacological interventions with broader psychosocial interventions such as IPS is outside the realm of NIMH support at this time. A test of whether pharmacological mechanisms combined with IPS may therefore have to wait for the approval of a medication for cognitive impairment associated with schizophrenia and funding by the owner of the medication.

## Other Pharmacological Augmentation Strategies

There have been several other domains of pharmacological interventions aimed at cognition in the schizophrenia spectrum, including interventions targeted at subclasses of monoamines, including dopamine D1 receptors and norepinephrine. D1 receptors have been an interesting target since animal work conducted by Patricia Goldman-Rakic et al. in the 1990s. Specifically, the D1 receptor agonist SKF 38393 was shown in several studies to have a direct beneficial effect on cognition in several animal models (71), although this compound does not cross the blood brain barrier in adequate concentrations to be useful for pharmacological augmentation. Other D1 agents, including dihydrexadine (DHX), have been tested in clinical trials. Although DHX did not lead to significant improvement in cognition in patients with schizophrenia (72), it was found to lead to significant improvements in working memory in patients with schizotypal personality disorder who had never been treated with antipsychotic medications (73). Broad spectrum dopamine agents such as pergolide (74) and pramipexole (75) have been examined in patients with schizotypal personality disorder and schizophrenia, respectively. However, pergolide has since been removed from the market because of adverse events, and pramipexole did not show remarkable efficacy in schizophrenia although it does not appear to have been tested in schizotypal personality disorder.

Similar findings were reported for the noradrenergic alpha-II agonist guanfacine, a currently approved treatment for attention deficit hyperactivity disorder found to improve cognition in animal models (76). In a clinical trial for schizophrenia, guanfacine did not improve cognition (77), but when used to treat patients with schizotypal personality disorder there was a significant positive treatment effect (78). Other noradrenergic interventions have also provided promising data, but have shown limited clinical efficacy in schizophrenia. For example, Friedman et al. (79) found that atomoxetine, a norepinephrine transport inhibitor, improved regional blood flow in critical areas compared to placebo in patients with schizophrenia; however, the cognitive benefits of the treatment did not achieve statistical significance. Kelly et al. (80) performed a 32-patient randomized trial and also did not find separation from placebo. However, all of these interventions have been tested only as monotherapy, without additional CRT or psychosocial interventions. The fact that cognition did not improve with these treatments does not mean that they would not manifest an incremental efficacy boost to these other learning-based interventions. As all of these treatments have demonstrated safety and tolerability, studies of combined therapy with these pharmacological strategies and CRT or psychosocial interventions would be able to proceed without many concerns about safety.

## Conclusion

Combining pharmacological interventions with CRT has been suggested as a promising way forward for improving cognition in schizophrenia and has been previously tested in randomized research trials. There are several different levels at which cognition-enhancing drugs could beneficially impact on the results of CRT, including making it easier for participants to engage in CRT (performance-side variables) as well as augmenting the extent to which these interventions have a benefit (increasing plasticity). Furthermore, a larger cognitive gain with combined therapy might also be more likely to lead to functional gains without the requirement for psychosocial interventions. Despite the substantial gains seen with some CRT interventions, including average effect sizes of *d* = 0.8, patients in those trials still have substantial cognitive deficits at the end of the study. If those effect sizes could be doubled, then cognitive performance could be normalized, which might have implications for whether additional skills training would necessarily be required.

The results of studies combining pharmacological and CRT interventions have been mixed, as a function of the mechanism of the pharmacological add-on strategy. It is possible that the research designs employed in unsuccessful studies were not optimal, such as those including single-dose treatments, medications that may actually impair learning or transfer of information, or concerns regarding the dosing of the CRT interventions delivered.

Much less mixed are the results of combining cognitive enhancement induced by CRT with psychosocial interventions. These studies have consistently found augmented rates of acquisition of psychosocial target outcomes in cases who received also CRT interventions. Finally, although in its infancy, another area where cognition-enhancing interventions could be explored is their potential ability to facilitate clinical gains in CBT, as per the single study that suggested CRT may lead to an incremental benefit for CBT. This would obviously be an area where a combined trial design could employ a pharmacological cognitive enhancer instead of CRT as a cognitive enhancement strategy to boost the efficacy of CBT.

We suggest that similar research strategies could also be employed with pharmacological augmentation, using a research design where a putative cognition-enhancing compound was added to psychosocial interventions in a randomized trial. There are currently limited data regarding this, but there are many promising agents to test. The pragmatics of pharmacological augmentation by-pass the effort and motivation-related limitations associated with some CRT interventions. This is a largely unexplored area, but the synergistic effect of psychosocial interventions and pharmacological treatment strategies seems worthy of exploration.

A final consideration is whether the triple combination of pharmacological, CRT, and psychosocial interventions would be superior to the results of combining CRT and psychosocial interventions alone. If pharmacological augmentation of CRT is actually beneficial and increases gains compared to CRT alone, then this combination might lead to even greater functional gains when combined with psychosocial interventions. This is, of course, a research question that can be addressed directly.

## Author Contributions

Both authors contributed equally to this article.

## Conflict of Interest Statement

In the past year, PH has served as a consultant to Allergan, Boehringer Ingelheim, Lundbeck, Otsuka Digital Health, Sanofi, Sunovion, and Takeda. MS is a full-time employee of Boehringer Ingelheim.
